# Ebola, fear and preparedness

**DOI:** 10.4178/epih/e2014015

**Published:** 2014-08-28

**Authors:** viroj wiwanitkit

**Affiliations:** Wiwanitkit House, Bangkhae, Bangkok, Thailand

Dear Editor,

The editorial on “Ebola, fear and preparedness” is very interesting [1]. As noted in the Editorial, “it is necessary for South Korea to make strategies to the outbreak by using present facilities as quickly as possible [1].” and “it is also imperative that the government establish suitable communication with its citizens to prevent the spread of uninformed fear and anxiety regarding the Ebola outbreak [1].” In fact, in any new emerging disease, the main problems are a) lack for information and knowledge for medical worker and b) sense of fear of panic for the general population [2]. To give the information to the general population in the early phase of outbreak can sometimes be difficult due to lack of data. The information might be too much or too little and it is necessary to update the information. Giving “fair information” to the general population is very important. To disguise fact should be avoided and this is the challenge for public health worker during epidemic. As noted by Davis et al. [3], “effective pandemic control requires a systematic dialogue with the publics.”At this phase, when the information is still incomplete, it is important to set up an information center to gathering all data and further analyzing and distributing it to governmental policy maker, health care worker and general population [4].
